# Food and Beverages Containing Algae and Derived Ingredients Launched in the Market from 2015 to 2019: A Front-of-Pack Labeling Perspective with a Special Focus on Spain

**DOI:** 10.3390/foods10010173

**Published:** 2021-01-16

**Authors:** Fatma Boukid, Massimo Castellari

**Affiliations:** Food Industries, Finca Camps i Armet s/n, Institute of Agriculture and Food Research and Technology (IRTA), 17121 Monells, Spain; massimo.castellari@irta.cat

**Keywords:** microalgae, macroalgae, carrageenans, food labeling, nutritional quality, market

## Abstract

Algae are a source of functional ingredients, with a large spectrum of healthy and functional compounds. Therefore, this study aimed to provide an overview on commercialized food and beverages made from algae and derived ingredients, with emphasis on the Spanish market, relying on the front-of-pack labeling. For this reason, the Mintel Global New Products Database was searched for foods and beverages containing “algae” ingredients, launched during the period 2015–2019. A total of 13,090 items were found worldwide, including 5720 items in Europe, in which 436 items were in Spain. Regardless of the market (global, European, and Spanish), a similar number of products categories (*n* = 20), dominant categories (dairy and desserts and ice cream) and dominant algal ingredient (carrageenans) were found. Nutritional information retrieved from Spanish products underlined that algae-based snacks had significantly lower energy, fat, and salt content compared to algae-free counterparts. On the contrary, spirulina- enriched ready to drink beverages had significantly higher energy and salt than algae-free. As such, reading the nutritional labeling is crucial to selecting products that suit consumer needs or/and expectations. Furthermore, only 8% of products reported the algal species and the level of inclusion, so this study emphasizes the importance of labeling legislation to provide complete product information to consumers.

## 1. Introduction

Algae are gaining popularity as “superfoods” across food and beverages categories, as main ingredients, flavoring agents, or natural colorants in premium-positioned launches [1,2,3,4]. Beside biodiversity and sustainability, algae are a valuable source of proteins, carbohydrates, phenols, vitamins, and minerals, depending on the species [5,6,7]. Algae can be divided into macroalgae, also known as seaweed, referring to macroscopic, multicellular and marine algae [brown (Phaeophyceae), red (Rhodophyceae), and green (Chlorophyceae)] [8], and microalgae referring to microscopic, freshwater, and marine unicellular species, including cyanobacteria, commonly known as blue-green algae, as well as green, dark colored, and red algae [9].

In the food industry, several prominent companies focus on the production and commercialization of food based on macroalgal biomass and products derived thereof. Macroalgae are promising sources of bioactive peptides and carbohydrates with potential beneficial biological activities (e.g., antihypertensive, anticoagulant, antioxidant, antiproliferative, and immunostimulatory) [5,10,11,12,13]. Furthermore, seaweed contains polysaccharides, such as alginate, carrageenans, and agar, which are commonly used as food hydrocolloids, providing relevant textural functionality (e.g., stabilizers, thickeners, and emulsifiers) in numerous processed foods [14,15,16,17]. The market for seaweed-derived hydrocolloids is governed by carrageenans, despite the controversial debates over its safety [18]. However, Food and Agriculture Organization of the United Nations and the World Health Organization (FAO/WHO) and Food and Drug Administration (FDA) established the safety of food additive carrageenans [19,20,21]. In the European Union (EU), carrageenan (E407) and processed Eucheuma seaweed (E407a) are authorized as food additives, according to the European Regulation (EU) No. 1333/2008 [22]. Over the last decade, the production of food products enriched with microalgae or compounds derived from microalgae has increasingly gained attention due to consumer awareness about their nutritious compositions and related health benefits [23,24]. Microalgae was used in reformulating several traditional products, including pasta [25,26], vegetable soup [27], bread [4,23,28,29], and snacks [30]. Most of these products contain either spirulina or chlorella, mainly because of their long history of use, while other species must apply to the Novel Food Regulation (EU) 2015/2283 before commercialization [31]. The global algae market was valued at USD $717.14 million in 2018 and is projected to reach USD $1365.8 million by 2027, at a compound annual growth rate (CAGR) of 5.35% between 2019 and 2027 [32]. This market includes a wide spectrum of applications, including food and beverages, nutraceuticals and dietary supplements, personal care, feed, pharmaceuticals, chemicals, and fuel. It is expected to grow at a compound annual growth rate of 4.2% from 2018 to 2025 [33]. The segment of food and beverages was the highest contributor to the global algae products market in 2017 and is projected to grow at a CAGR of 4.1%. In term of algal ingredients, the hydrocolloids segment accounted for 49.2% of algae product market share market and is expected to grow at a CAGR of 3.8% [34]. Likewise, the European market is growing with a CAGR of 5.2% in the forecast period of 2020 to 2027, and is expected to reach USD $1278.65 million by 2027 [35]. A similar trend was also observed in Asian (a CAGR of 6.2% from 2018 to 2025) and American markets (a CAGR of 6.25% from 2020 to 2025) [32]. The main drivers behind this market growth is the increasing demand for natural and healthy products to feed the growing populations, as well as the environmental and ethical concerns associated with animal products. Furthermore, as compared to plants food sources, algae do not require high land use, agricultural input, and freshwater. However, the complexity of the Novel Food Regulation for the commercial authorization of new products is one of the major factors limiting the expansion of the European algae sector [36].

In Spain, several companies are producing algae biomass including AgriAlgae^®^ (Madrid, Spain), AlgaeEnergy (Madrid, Spain), Green Sea Bio System One (Alicante, Spain), Algamoil Iberica S.L (Madrid, Spain), Aqualgae (Coruña, Spain), and Fitoplancton Marino S.L (Cadiz, Spain). Producers are focusing on improving strains selection, biomass production, cell disruption, and drying technologies, as well as reducing product cost. Since Spain possesses the optimal sun hours and intensity for microalgae cultivation, most companies are harvesting microalgae over macroalgae [37]. Although the current microalga biomass production cost per unit of dry biomass (3.4€ kg^−1^ at 100 ha scale) is relatively high, the high-value products (e.g., phycocyanin and carotenoids) are boosting the investment in this sector [37]. In food and beverages, the use of algae and derived ingredients reached 7.5% of total products launched in the Spanish market from 2015 to 2019, where Casa Ametller (Barcelona, Spain), Galletas Gullón (Palencia, Spain), Bionsan(Tarragona, Spain), and Grupo Dulcesol (Valencia, Spain) are among the important food producers [38]. In the future, the mitigation of off flavors related to algal ingredients will open more possibilities of applications and increase their addition level to product formulation to fully gain advantage of the nutritious composition of these ingredients. Moreover, veganism and vegetarianism trends will further urge manufactures to widen their product portfolios by formulating more innovative algae-containing products, such as meat analogues [39].

Concern over the nutritional value of food and beverages and increased awareness over the association between health and nutrition have a major impact on consumer acceptance of using algae as a food source. The first tool for delivering the nutrition and health information to consumers is the food label. European Regulation (EU) No. 1169/2011 regulates the compulsory information mentioned in the labeling of foods, including the list of ingredients and the nutritional declaration [40]. The Labeling Directive requires manufacturers to declare all ingredients present in pre-packaged foods sold in the EU, with very few exceptions, to avoid consumer misleading. In the past, several research studies reported that some commercial products did not comply with the legislation, or what was listed on the label did not correspond to the actual product [41,42]. This suggests paying more attention to food labeling, food composition, and food claims.

Due to the nutritional composition of algae, consumers can be attracted to purchase specific foods and beverages simply because they are assuming the healthiness of these products, since their list of ingredients contain “algae” [1], without checking the complete nutritional information. A recent study showed that EU consumers prefer meat substitutes based on microalgae that are organic and local [43]. This means that communicating the health benefits of microalgae on the label has the potential to increase consumer choice. Price also plays an important role for consumers. For instance, local and organic labeled foods require higher prices, yet communicating the environmental-friendly algae production might further increase consumer preference. All details mentioned on the label are extremely important to consider for sellers, manufacturers, and consumers, as well as policy makers. In this light, the present work has a dual objective: (i) to enable a general overview about global and European markets of food and beverages categories formulated with “algae and derived ingredients”, and (ii) to focus on the Spanish market as a case study to investigate the impact of “algae and derived ingredients” on the nutritional profile of new food and beverages.

## 2. Materials and Methods

### 2.1. Data Collection

Identification of new food and beverages launched worldwide in the last five years (2015–2019) was carried out by consulting the Mintel Global New Product Database (Mintel GNPD-Mintel Group Ltd., London, UK). The Mintel GNPD tracks packaged food and beverage launches in 86 markets worldwide. Each item has detailed product information, such as price, ingredients, claims made and nutritional information, as well as photographs of all sides of the packaging.

New food and beverage products launched in the period 2015–2019 were searched for; products containing algae or its derived ingredients using the keyword “Algae and all child ingredients” were used to select all algae derived ingredients. The Mintel GNPD search was conducted on 17 April, 2020, using the search parameters listed in Table 1. This search was performed for the global market, European market, and Spanish market using the same parameters. The results of all searches were exported to Microsoft Excel (Microsoft Office, Washington, WA, USA).

Mintel GNPD classifies foods in several categories (baby food, bakery, breakfast cereal, chocolate confectionery, dairy, desserts and ice cream, fruits and vegetables, meals and meal centers, processed fish, meat and egg products, sauces and seasonings, savory spreads, side dishes, snacks, soup, sugar, and gum confectionery) as well as beverages (carbonated soft drinks, hot beverages, juice drinks, sports and energy drinks, ready to drink beverages—RTD, water and other beverages). Some of these categories were excluded from the search as they were inappropriate for this study (Table 1).

Another search was conducted on Mintel to determine the nutritional composition of algae-free snacks and ready-to-drink beverages (RTDs) launched in the Spanish market in the last five years, to compare the nutritional profile of the algae-based products and their algae-free counterparts.

### 2.2. Data Extraction

For products launched in the Spanish market, nutritional information (energy (kcal/100 g or 100 mL), total fat (g/100 g or 100 mL), saturated fatty acids—SFA (g/100 g or 100 mL), carbohydrates (g/100 g or 100 mL), sugars (g/100 g or 100 mL), protein (g/100 g or 100 mL), and salt (g/100 g or 100 mL)), as well as algae (strain and level of addition) or derived ingredients, were retrieved.

### 2.3. Statistical Data Analysis

The statistical analysis was carried out using the Statistical Package for Social Sciences software (IBM SPSS Statistics, Version 25.0, IBM corp., Chicago, IL, USA). Based on Kolmogorov–Smirnov test; the normality of data distribution was rejected, and therefore data were expressed as median values with interquartile ranges. Energy and nutrient contents per 100 g or 100 mL of products were analyzed using Kruskal–Wallis non-parametric one-way ANOVA for independent samples with multiple pairwise comparisons and Mann–Whitney non-parametric test for two independent samples.

## 3. Results

### 3.1. Categorization of New Food and Beverages Containing Algal Ingredients Launched in the Global and European Market (2015–2019)

During the period 2015–2019, 13,090 new products (79.0% foods and 21.0% beverages) containing algal ingredients (raw biomass or extracts) were released worldwide (Figure S1). The most represented food categories were dairy (*n* = 4444, 33.9%) and desserts and ice cream (*n* = 2404, 18.4%). These products were mainly launched in Europe (*n* = 5720; 43.7%) followed by Asia Pacific (*n* = 3209; 24.5%), North America (*n* = 1983; 15.1%), Latin America (*n* = 1767; 13.5%), Middle East and Africa (*n* = 411; 3.1%). In terms of algal ingredients, carrageenans (carrageenan E 407 and processed Eucheuma seaweed E 407a) were the most included in the formulation of new products launched globally in the period 2015–2019 (*n* = 10428; 79.7%), followed by spirulina (*n* = 552; 4.2%), “other algae” (with no specification of the strain), spirulina concentrate (*n* = 456; 3.5%) and *Lithothamnium calcareum* (*n* = 272; 2.1%).

In the European market, the 5720 new products containing algal ingredients launched were mainly foods (80.2%) and, to a lesser extent, beverages (19.8%). The most relevant categories were desserts and ice cream (*n* = 1640; 28.7%) and dairy (*n* = 1396; 24.4%) (Figure S1). In the EU market, carrageenans lead the list of algal ingredients (*n* = 4350; 76.0%) followed by spirulina (*Arthrospira* sp.) concentrate (*n* = 401; 7.0%), spirulina powder and *Lithothamnium calcareum* (*n* = 228; 4.0%), and other unidentified “algae” (*n* = 224; 3.9%). For the remaining products, other algal strains were declared including *Chlorella* sp., kelp (*Laminariales*), nori (*Porphyra* spp.), Wakame seaweed (*Undaria pinnatifida*) and Kombu seaweed (*Laminaria ochroleuca*).

### 3.2. Categorization of New Food and Beverages Containing Algal Ingredients Launched in the Spanish Market (2015–2019)

In the Spanish market, a total of 436 new items containing algal ingredients were launched during the period 2015–2019 (84.2% foods and 15.8% beverages). In this case the most represented categories were desserts and ice cream (*n* = 91; 20.9%), processed fish, meat and egg products (*n* = 67; 15.4%) and snacks (*n* = 24; 5.5%) (Figure S1). In term of ingredients, carrageenans were the most used algae-based ingredient (*n* = 332; 76.1%) included in the in new products launched in the Spain market in the period 2015–2019, followed by *Lithothamnium calcareum* (*n* = 22; 5.0%), spirulina (*n* = 18; 4.1%), chlorella (*n* = 13; 3.0%), Wakame seaweed (*n* = 13; 3.0%), spirulina concentrate (*n* = 12; 2.8%), Kombu seaweed (*n* = 10; 2.3%), and other unidentified algae-based ingredients (*n* = 10; 2.3%).

### 3.3. Nutritional Properties of Algae-Based New Products Launched in Spain

Out of all identified food and beverages items (*n* = 436), products with no nutritional labeling were excluded. These products did not have quantitative ingredients declaration as they are foods consisting of a single ingredient (dried algae) and, therefore, the quantity of single ingredients corresponds to 100% based on the commission notice on the application of the principle of quantitative ingredients declaration (QUID) (2017/C 393/05) [44]. As a result, 97% (*n* = 425 items) of the products were retrieved. Among these items, only in 8% of the products (*n* = 34 items), the amount of algae-based ingredient was reported in the packaging, namely for spirulina (from 0.1% to 62% addition level), chlorella (0.1 to 0.5% addition level) and *Lithothamnium calcareum* (0.4 to 0.5% addition level). The absence of quantitative declaration can be attributed to the use of “small quantities” of algae for the purposes of flavoring based on the QUID (2017/C 393/05). Nevertheless, the term “small quantities” is not clarified or specified in the QUID.

The compulsory nutrition information indicated by Council Regulation (EU) No. 1169/2011 [40] printed on the packaging of the 425 products is reported in Table S1. Table 2 summarizes the dataset as a function of categories of products. As expected, significant differences were found among food and beverages categories in terms of nutritional profile. Breakfast cereals and chocolate confectionery showed the highest median energy content (514 kcal/100 g and 477 kcal/100 g, respectively) followed by snacks (357 kcal/100 g), side dishes (357 kcal/100 g) and bakery (345 kcal/100 g). Likewise, breakfast cereals and chocolate confectionery also had the highest contents of total and saturated fats (24.8 g/100 g and 16 g/100 g, respectively; 21.2 g/100 g and 9.6 g/100 g, respectively). Sugar and gum confectionery and side dishes recorded the highest total carbohydrate content followed by breakfast cereals, chocolate confectionery and bakery. Chocolate confectionery showed the highest contents of sugar (62.3 g/100 g) followed by breakfast cereals, bakery and desserts and ice cream. As for proteins, processed fish, meat and egg products had the highest values (13.5 g/100 g) similarly to snacks and other beverages. Salt content ranged from 0.0 g/100 g (juice drinks) to 1.7 g/100 g (processed fish, meat and egg products).

To have a clearer picture about the nutritional impact caused by the inclusion of algal ingredients in reformulated food products, the nutritional profiles of snacks and RTDs reformulated with raw algal biomass (e.g., whole seaweeds or microalgal single cell powder, not considering extracts containing carrageenans) were compared to those of the corresponding new products not including algal ingredients (algae-free) launched in the Spanish market in the last five years. Only two food categories, snacks and ready to drink beverages (RTDs), were included in this comparison because they were the only ones reformulated exclusively with raw algal biomass (i.e., from nori, chlorella and spirulina) and these two categories were numerous enough to carry out a suitable statistical analysis (snacks *n* = 29; RTDs *n* = 20). The median values of nutritional characteristics of algae-free products (snacks and RTDs) are reported in the Supplementary Materials (Tables S2 and S3).

**Figure 1:**
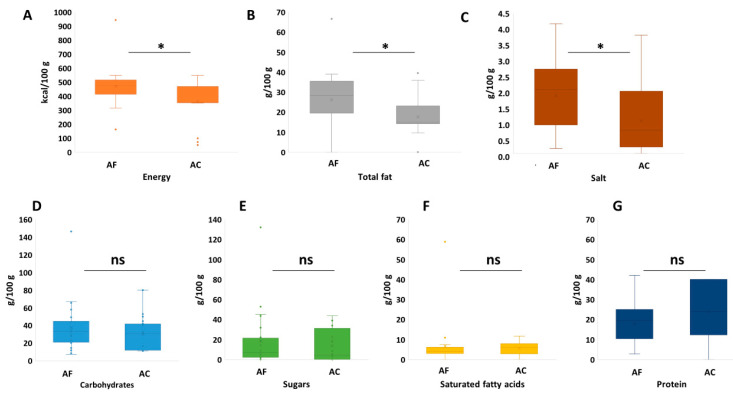
Nutritional profile of snacks formulated with algae (*n* = 24) vs. algae-free snacks (*n* = 24). (**A**): energy (kcal/100 g), (**B**): total fat (g/100 g), (**C**): salt (g/100 g), (**D**): carbohydrate (g/100 g), (**E**): sugars (g/100 g), (**F**): saturates (g/100 g), and (**G**): protein (g/100 g). AF: algae-free and AC: algae-containing; statistical significance based on Kolmogorov–Smirnov test (*: *p* < 0.05, ns: non-significant (*p* > 0.05)); the box-plot legend: the box is limited by the lower (Q1 = 25th) and upper (Q3 = 75th) quartile; the median is the horizontal line dividing the box; Whiskers above and below the box indicate the 10th and 90th percentiles; outliers are the points outside the quartile 10–90th percentiles.

In the case of snacks, the reformulated products were enriched with nori (*n* = 9), chlorella (*n* = 9) and spirulina (*n* = 11). For these products, no significant difference among the nutritional properties as a function of species could be evidenced. So, the statistical comparison was carried out between the products reformulated with algae and the algae-free ones, without considering the algal origin. The statistical analysis of the nutritional parameters (Figure 1) showed that no significant difference could be evidenced between algae-free and algae-containing snacks for carbohydrate, sugar, saturated fats, and protein contents. Moreover, algae-free snacks had significantly higher energy, fat, and salt than algae-based snacks.

**Figure 2:**
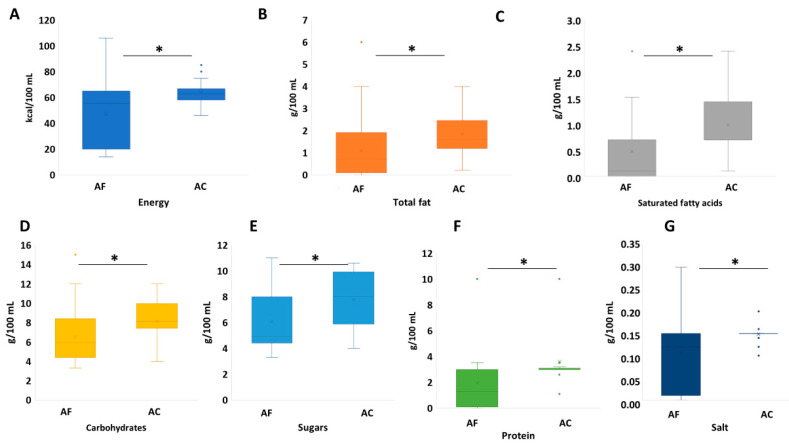
Nutritional profile of ready to drink beverages (RTDs) formulated with algae (*n* = 20) vs. algae-free RTDs (*n* = 51) in terms of nutritional information. (**A**): energy (kcal/100 g), (**B**): total fat (g/100 mL), (**C**): saturates (g/100 mL), (**D**): carbohydrate (g/100 mL), (**E**): sugars (g/100 mL), (**F**): protein (g/100 mL) and (**G**): salt (g/100 mL), (**A**–**F**): algae-free and (**A**–**C**): algae-containing; statistical significance based on Kolmogorov–Smirnov test (*: *p* < 0.05, ns: non-significant (*p* > 0.05)); the box-plot legend: the box is limited by the lower (Q1 = 25th) and upper (Q3 = 75th) quartile; the median is the horizontal line dividing the box; Whiskers above and below the box indicate the 10th and 90th percentiles; outliers: are the points outside the quartile 10–90th percentiles.

In the case of RTDs, all algae-containing products (*n* = 20) were formulated with spirulina. As illustrated in Figure 2, algae enriched RTDs had significantly higher median values for all the nutritional parameters included in this study when compared to the corresponding algae-free products.

## 4. Discussion

The use of algae in food formulation is positioning firmly in the food market. Earlier, algae ingredients were mostly used as dietary supplements in different forms, such as powder, capsules, and tablets due to their rich composition in health beneficial compounds (e.g., carotenoids, astaxanthin, omega-3, and docosahexaenoic acid) [45,46]. Recently, the trend is the use of these ingredients (or deriving ingredients) in food formulations. The number of food and beverage launches containing microalgae or macroalgae has significantly increased during the past five years [1]. During the period 2015–2019, 13,090 new food products containing algae or derived components were globally launched, of which 5720 were launched in Europe, and 436 in Spain. The algae incorporation took different forms depending on the ingredient used (whole dried biomass or a purified ingredient) and their function in the formulation (coloring agent or functional ingredient) [24]. This versatility of use offers plenty of opportunities for food manufacturers and, at the same time, it can be quite confusing for the consumer when choosing an algae-containing product over algae-free products. Several factors can impact consumer choices, including the nutritional labeling and the mention of algae strain on the label. The questions that can be raised are: for what reasons are algae used as food ingredients? Are their uses attributed more to their nutritional and functional properties or as a marketing strategy? The label plays a crucial role in consumer selection [47]. Noteworthy, the perception from consumers concerning algae-based products and their willingness to pay are not static, but continuously changing, depending on consumer awareness on the health benefits of these ingredients, environmental impact and sustainability concerns, as well animal/fish welfare [43]. Most consumers associate algae with healthy food [48]. Furthermore, nutrition and health claims further impact consumer choice, as well as other labels such as organic, natural, local, vegetarian, and vegan. Based on recent market reports, algae market demand is witnessing continuous growth, owing to the high nutritional value of algal ingredients, the expansion of the vegan population, and the increasing demand for plant-based food products [33,49].

In the last five years, carrageenans were identified as the most used algal ingredients in the formulation of new food products launched globally, in the EU, and in Spanish markets (79.7%, 76.0%, and 76.1%, respectively), while other algal ingredients (e.g., raw seaweed or microalgal single cell products) were included to a lesser extent. In one product launched in the Spanish market (extra cooked ham), both E 407 and E 407a were used simultaneously in the formulation. It should be noted that the European Food Safety Authority (EFSA) reported that E 407 and E 407a are not likely to be used in combination in the same food product [36]. Carrageenans have a long history of use as seaweed-derived hydrocolloids offering thickening, stabilizing, and gelling functionalities for a multitude of food applications [18,50].

Products containing seaweed ingredients accounted only for 3.96% (*n* = 516) of the new food products containing algal ingredients launched in the global market in the last five years (2015–2019), showing a stable spread if compared to the period 2011–2015 [37]. Macroalgae have been well recognized for centuries in Asian (e.g., Japan and Korea) diets, as a great source of complex polysaccharides, minerals, proteins, and vitamins, as well as several bioactive components [51]. The use of seaweed as a food ingredient is increasing in the European market at an exponential rate and it is classified as the most innovative market; after that, the Asian market [52], reaching 4.73% (*n* = 270) of total products containing algae ingredients. In Spain, their use is more relevant (12.10%; *n* = 53) due to the high number of annually launched innovative products.

Globally, the use of microalgae accounted for 11% of total products, where spirulina was the most used species, similar to the European and Spanish markets. Spirulina shows high potential to gain a higher share of the market in the future and to be used as an ingredient (as a dietary supplement or whole food) in the development of wider spectrum of functional foods [53]. Chlorella is used at a lesser extent in food products since its main application is in the nutraceutical and pharmaceutical sectors [54]. In the EU, spirulina is widely used as a food ingredient in launched products (13.8%, *n* = 791) and promoted as a “superfood” since these two species have a long history of use and are approved for use in food products in the EU [23,27]. In Spain, the use of microalgae-based ingredients significantly increased during the past 5 years to reach 11.2% of total launched products, where spirulina-based items raised from 1.6% (2015) to reach 5.4% (2019), as well as chlorella (from 0% in 2015 to 5.4% in 2019), owing to increasing awareness about their health benefits and sustainability [24]. Hence, these characteristics, in combination with appropriate food labeling, have large potential to increase the market share of products containing algal ingredients, since their marketing strategy is primarily based on their “healthy” images [43]. Furthermore, the therapeutic applications (e.g., hypolipidemic, antioxidant, and anti-inflammatory) of spirulina and chlorella will further boost their application, but going through the European Food Safety Authority (EFSA) procedure is required prior to claim such health benefits on the labels.

Although the level of inclusion of algal ingredients directly influences the nutritional profile of the reformulated products, only 8% of all new products launched in the Spanish market in the latest five years contained this information on the package. In this frame, the inclusion of algal ingredients, at very low percentages, could be mainly driven by marketing reasons, to take advantage of consumer awareness about microalgal health benefits, even if it could not generate a significant change in the nutritional profile of the product [23,55,56]. The Regulation (EU) No 1169/2011 requires the indication of the quantity of certain ingredients or categories of ingredients used in the manufacture or preparation of all prepacked foods, but there is no obligation to declare when “small quantities” were used [44].

In most cases, the use of algal biomass (both macroalgae and microalgae) in food products was at low concentrations, ranging from 0.3% to 1.5%, such as in pasta, soups (from 0.9 to 1.5% Wakame seaweed) or dairy (0.3–0.5% *Lithothamnium calcareum*) or meals and meal centers (0.5% of nori). Several studies showed that the inclusion of algal ingredients in food matrices at high levels could be extremely challenging due to their negative impact on the sensorial rheological properties that the algal ingredients may induce on the final product, such as soups and creams, bread, and pasta [27,57]. In chocolate confectionery, a higher level (4%) of Wakame seaweed was used probably because the strong flavor of cacao partially masks the macroalgae taste. Seaweed-based chocolates were found with acceptable sensory properties and reported as a good source of iron (56 mg of iron/100 g and 11.80 mg of bioavailable iron) causing a significant increase in hemoglobin, serum iron and serum ferritin levels for anemic adolescent girls [58]. On the other hand, spirulina was used as a main ingredient in snacks (up to 62% of inclusion) and beverages (up to 25% of inclusion) showing that these products are suitable vehicles to deliver the nutritionally valuable algal components, such as essential fatty acids, essential amino acids, phycocyanin, and carotenoid, in a versatile and convenient presentation [30]. Although microalgae have distinctive characteristics (color, flavor, and odor), consumers gave good sensorial scores, considering that these products are healthier snacks [59].

The results of our comparative study showed that algae-based snacks launched in the Spanish market in the last five years had lower energy and fat and salt content compared to the corresponding algae-free products. In general, the inclusion of algal ingredients did not significantly vary the protein content if compared to the algae-free products, probably due to the high variability in the amounts of added algae (spirulina, nori, and chlorella) in the new formulations (when the level of addition was reported, values ranged from 0.15% to 62%). For instance, snacks enriched with 0.15% spirulina contained 0.7 g/100 g protein; while those made with 62% spirulina had 25 g/100 g proteins. Otherwise, results confirming the nutritional impact of spirulina on snacks were reported in several works [27,60]. The addition of 2.5% spirulina resulting in an increase of 22.6% in proteins, 28.1% in lipids, and 46.4% in minerals compared to those spirulina-free [30]. This addition did not hinder the technological and physical properties of the products, and they were found acceptable after sensory analysis. Consistently, it was reported that the addition of 2% and 6% of spirulina increased protein by 11.7% and 29.9%, respectively [61]. However, the increasing level of inclusion was related to decreased sensorial acceptance of these products.

Algae-free RTDs (*n* = 51) showed a higher intra-variability of their nutritional profile compared to those reformulated with algal ingredients (*n* = 20), which were exclusively enriched with spirulina. The high variability in RTDs were also found in surveys conducted in different countries (France and Romania) due to the diversity of ingredients (fruits and vegetables) and taste (sweet, regular tonics) [62]. In all cases, the percentage of inclusion of spirulina was not reported, which makes difficult the interpretation of the increase of energy, carbohydrates, fat and salt in these products. In turn, the higher protein content in algae enriched RTDs can be probably attributed to the inclusion of the microalgal ingredient in their formulation.

## 5. Conclusions

The present work was conceived in the frame of the ProFuture project, and its primary objective was, firstly, the categorization of food and beverages containing algae and derived ingredients launched in the last five years in the global market, as well as the analysis of the nutritional quality of commercial food and beverages containing algal ingredients launched in the last five years (2015–2019) in the Spanish market.

Global, European, and Spanish markets presented many similarities in terms of dominant food categories enriched with algae and derivatives thereof. In particular, carrageenan was the predominant algae-derived ingredient in the market, while single-cell algal ingredients are still included in the new formulations, to a lesser extent. Considering the Spanish market as a case study, the present work noted significant differences between the nutritional profiles of recently launched algae-enriched and algae-free snacks and RTDs.

This last part of the study was affected by some limitations, which make difficult the assessment of the nutritional impacts derived from the reformulation with algae, because: (i) only around 24% of total items were actually reformulated with whole macroalgae or microalgae, while the rest of the products only contained carrageenan; (ii) 92% of products did not include the level of addition on the package; and (iii) the evaluation of nutritional quality focused exclusively on mandatory information and excluded fiber, vitamins, minerals, and bioactive compounds.

Even so, this study provides a preliminary indication about the nutritional profile of the new products (algae-enriched) available in the Spanish market, as it covers all categories and subcategories of products using Mintel GNPD, and focused on launches from the last five years to enable a more complete picture of market evolution. Algae-based snacks launched in Spain in the latest five years provided lower energy, fat, and salt, but not more protein than the algae-free analogous products. On the contrary, RTDs reformulated with algal ingredients showed increased levels of protein compared to their algae-free counterparts, but energy, total and saturated fats, carbohydrates, sugar, and salt also increased significantly.

So, from a nutritional point of view, the reformulation of food and beverages with algal ingredients does not automatically guarantee improvement of the nutritional profile if compared to the corresponding algae-free products. It is also noteworthy that microalgal single cell ingredients are generally included at very low percentages in reformulated products, so there is still a huge untapped opportunity to incorporate them in food and beverages. In this sense, more research and innovation is required to boost the development of purified, organoleptically, more neutral, algal ingredients, which could be likely included at higher percentages in food and beverages, and which could help to better tailor the optimization of the nutritional profile, allowing increased percentages of inclusion without altering the organoleptic properties. Overall, the results showed that informative labeling and a more precise declaration of algae ingredient (strain and level) on the labels are deemed necessary to help consumers choose algae-enriched products in a conscious way.

## Figures and Tables

**Table 1:** Search strategy used on Mintel Global New Product Database.

Search Variables	Parameters
Country	First search: worldwide Second search: Europe Third search: Spain
Date published	Last five years: 2015–2019
Ingredient search	Algae and deriving ingredients
Mintel GNPD categories included	Food: desserts and ice cream, processed fish, meat and egg products, snacks, meals and meal centers, bakery, sauces and seasonings, soup, baby food, chocolate confectionery, fruit and vegetables, savory spreads, sugar and gum confectionery, breakfast cereals, side dishes, sweet spreads, sweeteners and sugar, Drink: other beverages, ready to drink beverages (RTDs), juice drinks, hot beverages, sports and energy drinks, carbonated soft drinks
Mintel GNPD categories excluded	Alcoholic beverages, water

**Table 2:** Nutritional composition of food and beverages launched in the Spanish market in the latest 5 years containing algae and derived ingredients.

	N	Energy kcal/100 g or 100 mL	Total Fat g/100 g or 100 mL	Saturates g/100 g or 100 mL	Carbohydrate g/100 g or 100 mL	Sugars g/100 g or 100 mL	Protein g/100 g or 100 mL	Salt g/100 g or 100 mL
**Desserts and Ice Cream**	91	227(93–312) ^abc^	9.6(2.1–15.4) ^abc^	6.1(0.7–10.8) ^abc^	22.8(15.2–33) ^abc^	20.6(11.8–27) ^bc^	3.4(2.9–4) ^ab^	0.2(0.1–0.2) ^a^
**Bakery**	9	345(287–413) ^abc^	16(7.2–19.7) ^abc^	2(1.5–7.7) ^abc^	47(39.5–56.3) ^de^	24(20–32) ^cd^	5.9(5.1–7) ^ab^	0.8(0.5–1.4) ^a^
**Baby Food**	5	79(70.5–81.5) ^ab^	2.7(2.2–3) ^ab^	0.8(0.2–1) ^a^	11.4(9.8–11.4) ^abc^	7.8(7.4–8.2) ^abc^	1.9(1.9–2.9) ^a^	0.1(0.1–0.1) ^a^
**Sugar and Gum Confectionery**	4	241(227.5–317.4) ^cd^	0.1(0–1.5) ^ab^	0.1(0–0.3) ^a^	95(80.3–97.8) ^f^	0.3(0–31.6) ^abc^	0.2(0–4.6) ^a^	0.1(0–0.1) ^a^
**Breakfast Cereals**	3	514(380-ND) ^d^	24.8(2.7-ND) ^d^	16(1.3-ND) ^c^	65(65-ND) ^ef^	43(31-ND) ^cd^	6(6-ND) ^abc^	0(0-ND) ^a^
**Processed Fish, Meat, and Egg Products**	61	124(98–176) ^ab^	4(2–9.1) ^abc^	0.9(0.6–1.6) ^a^	5.5(1.9–12) ^ab^	1.5(0.5–2) ^a^	13.5(11–18.2) ^abc^	1.7(1.2–2) ^a^
**Savory Spreads**	4	160(83–327) ^abc^	11.2(2.7–31.2) ^bcd^	2.2(0.3–10.2) ^abc^	5.2(2.7–12.7) ^a^	0.5(0.3–1.1) ^a^	9.2(3–9.8) ^abc^	1.6(0.8–2.3) ^a^
**Side Dishes—pasta**	2	357(355-ND) ^cd^	2.2(2-ND) ^ab^	0.7(0.6-ND) ^a^	69(68-ND) ^e^	4(0.2–7.8) ^ab^	12(1.1–2.7) ^abc^	0.2(0.2-ND) ^a^
**Sauces and Seasonings**	6	159(130–197) ^ab^	15.6(10.6–18) ^abcd^	3.3(2.1–12) ^abc^	6.5(1–7.5) ^a^	3.9(1.7–3.2) ^ab^	1.8(4.6–10) ^a^	0.9(0.4–2.9) ^a^
**Meals and Meal Centers**	14	191(153.5–205.25) ^abc^	6.9(6.2–8.6) ^abc^	3(1–4.3) ^ab^	16.6(13.5–25.2) ^abc^	2.3(37.5–73) ^ab^	5.6(2.4–9.3) ^abc^	1.2(0.8–1.8) ^a^
**Chocolate Confectionery**	6	477(460–561) ^d^	21.2(16.3–38.6) ^d^	9.6(8.8–18) ^c^	64.3(39.9–73.2) ^e^	62.3(0.1–31.1) ^e^	3.1(12.4–40) ^abc^	0.2(0.2–5.7) ^ab^
**Snacks**	24	357(353–470.25) ^cd^	15(14.3–23.2) ^cd^	6.2(3–7.9) ^abc^	31(12–41.6) ^bcd^	4.5(0.1–1.9) ^abc^	24(0.7–1.7) ^bc^	0.7(0.2–2) ^a^
**Fruit and Vegetables**	5	21(16.285–27.15) ^a^	0.3(0.3–1.5) ^ab^	0.1(0–0.2) ^a^	0.7(0.3–2.1) ^a^	0.5(5.9–9.9) ^a^	1.2(3–3.1) ^a^	1(0.3–2.1) ^a^
**RTDs**	20	63(58–66.75) ^a^	1.6(1.2–2.5) ^ab^	0.7(0.7–1.4) ^a^	8.1(7.4–10) ^abc^	8(4–10) ^abc^	3(1.8–3.7) ^abc^	0.2(0.2–0.2) ^a^
**Dairy**	114	68(56–99.75) ^ab^	1.6(1.1–3.1) ^abc^	0.6(0.3–1.4) ^a^	9.2(5.5–12) ^abc^	7.2(7.3–12) ^abc^	3(0.3–0.9) ^a^	0.1(0.1–0.2) ^a^
**Juice Drinks**	17	56(43.5–59.5) ^a^	0.1(0–0.8) ^a^	0(0–0.4) ^a^	11.9(8.7–12.8) ^abc^	10(1.1–9.2) ^abc^	0.4(1.3–12.8) ^a^	0(0–0.1) ^a^
**Soup**	8	278(28.25–354) ^abc^	2.3(0.6–12) ^abc^	0.3(0.1–1) ^a^	38(5–53.1) ^cd^	2.5(1.5–27) ^ab^	9.1(0–4.6) ^abc^	1.4(0.8–13.3) ^b^
**Hot Beverages**	5	120(20–187) ^ab^	3.4(1.5–5.2) ^abc^	2.3(1.5–3.3) ^abc^	12(4–32.9) ^abc^	12(1.1–7.4) ^abc^	3.3(5–29.6) ^ab^	0.1(0–0.3) ^a^
**Other Beverages**	24	142(65–332.375) ^abc^	1.8(1.3–4.8) ^abc^	0.6(0.3–1) ^a^	8.9(4.1–20.1) ^abc^	3.2(0–0.1) ^ab^	9.2(0.1–0.2) ^c^	0.3(0.1–0.4) ^a^
**Sports and Energy Drinks**	1	226	0	0	51	3.7	0.5	0.1

Values are expressed as median (25th–75th percentile). ND: not determined; N: number of items. For each nutritional characteristic, different superscript letters in the same column indicate significant differences among type (Kruskal–Wallis non-parametric one-way test for independent samples with multiple pairwise comparisons), *p* < 0.05.

## Supplementary Materials

The following are available online at https://www.mdpi.com/2304-8158/10/1/173/s1, Figure S1: Categorization of food and beverages, containing algae and derived ingredients, launched from 2015 to 2019; Table S1: Nutritional profile of products with algae ingredients launched in the last five years and commercialized in the Spanish market; Table S2: Nutritional profile of snacks without algae launched in the last five years and commercialized in the Spanish market; Table S3: Nutritional profile of ready to drink (RTD) beverages without algae launched in the last five years and commercialized in the Spanish market
