# Supplementary material for: Food and Beverages Containing Algae and Derived Ingredients Launched in the Market from 2015 to 2019: A Front-of-Pack Labeling Perspective with a Special Focus on Spain
Source: Foods. 2021 Jan 16;10(1):173. doi: 10.3390/foods10010173 (PMC7830845; doi:10.3390/foods10010173)
Supplement: Supplementary file 1 [file foods-10-00173-s001.zip › Supp Materials/Figure S1.pptx]

## Slide 1
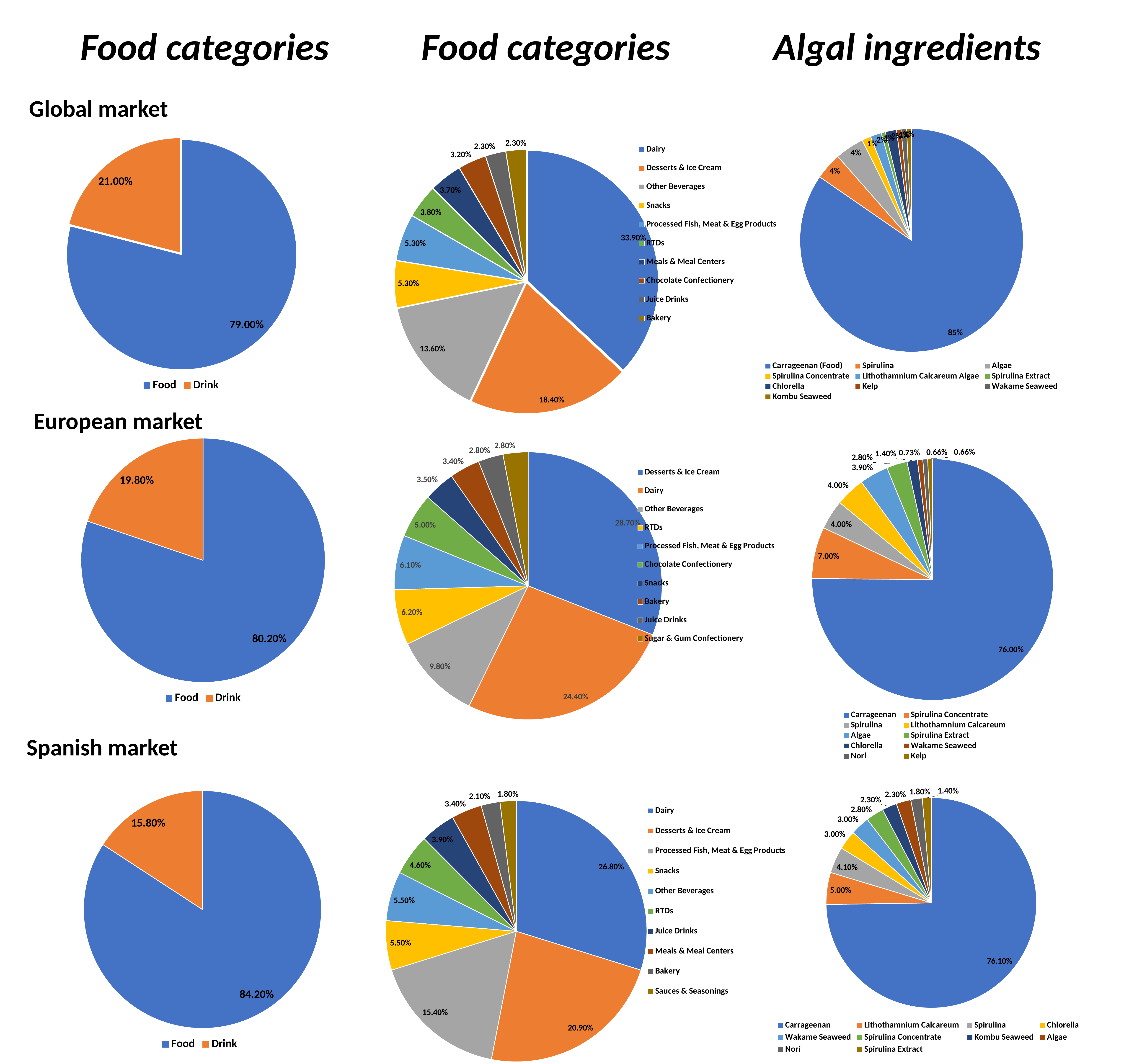

Food categories
Food categories
 Algal ingredients
Global market
### Chart
| Category | |
|---|---|
| Carrageenan (Food) | 0.846 |
| Spirulina | 0.039 |
| Algae | 0.042 |
| Spirulina Concentrate | 0.013 |
| Lithothamnium Calcareum Algae | 0.016 |
| Spirulina Extract | 0.0061 |
| Chlorella | 0.016 |
| Kelp | 0.0066 |
| Wakame Seaweed | 0.008 |
| Kombu Seaweed | 0.0071 |
### Chart
| Category | |
|---|---|
| Food | 0.79 |
| Drink | 0.21 |
### Chart
| Category | |
|---|---|
| Dairy | 0.339 |
| Desserts & Ice Cream | 0.184 |
| Other Beverages | 0.136 |
| Snacks | 0.053 |
| Processed Fish, Meat & Egg Products | 0.053 |
| RTDs | 0.038 |
| Meals & Meal Centers | 0.037 |
| Chocolate Confectionery | 0.032 |
| Juice Drinks | 0.023 |
| Bakery | 0.023 |European market
### Chart
| Category | |
|---|---|
| Food | 0.802 |
| Drink | 0.198 |
### Chart
| Category | |
|---|---|
| Desserts & Ice Cream | 0.287 |
| Dairy | 0.244 |
| Other Beverages | 0.098 |
| RTDs | 0.062 |
| Processed Fish, Meat & Egg Products | 0.061 |
| Chocolate Confectionery | 0.05 |
| Snacks | 0.035 |
| Bakery | 0.034 |
| Juice Drinks | 0.028 |
| Sugar & Gum Confectionery | 0.028 |
### Chart
| Category | |
|---|---|
| Carrageenan | 0.76 |
| Spirulina Concentrate | 0.07 |
| Spirulina | 0.04 |
| Lithothamnium Calcareum | 0.04 |
| Algae | 0.039 |
| Spirulina Extract | 0.028 |
| Chlorella | 0.014 |
| Wakame Seaweed | 0.0073 |
| Nori | 0.0066 |
| Kelp | 0.0066 |Spanish market
### Chart
| Category | |
|---|---|
| Carrageenan | 0.761 |
| Lithothamnium Calcareum | 0.05 |
| Spirulina | 0.041 |
| Chlorella | 0.03 |
| Wakame Seaweed | 0.03 |
| Spirulina Concentrate | 0.028 |
| Kombu Seaweed | 0.023 |
| Algae | 0.023 |
| Nori | 0.018 |
| Spirulina Extract | 0.014 |
### Chart
| Category | |
|---|---|
| Food | 0.842 |
| Drink | 0.158 |
### Chart
| Category | |
|---|---|
| Dairy | 0.268 |
| Desserts & Ice Cream | 0.209 |
| Processed Fish, Meat & Egg Products | 0.154 |
| Snacks | 0.055 |
| Other Beverages | 0.055 |
| RTDs | 0.046 |
| Juice Drinks | 0.039 |
| Meals & Meal Centers | 0.034 |
| Bakery | 0.021 |
| Sauces & Seasonings | 0.018 |
